# Supplementary material for: Vaccine Production Process: How Much Does the General Population Know about This Topic? A Web-Based Survey
Source: Vaccines (Basel). 2021 May 29;9(6):564. doi: 10.3390/vaccines9060564 (PMC8229207; doi:10.3390/vaccines9060564)
Supplement: Supplementary file 1 [file vaccines-09-00564-s001.zip › vaccines-1184837-supplementary.pdf]

vaccines

MDPI

Supplementary Materials

File S1: Informed Consent, Questionnaire and Tables

INFORMED CONSENT (BLANK FORM)

The aim of this survey is collecting information about general population's doubts concerning vaccine production process, in order to understand how this topic may influence the vaccine hesitancy.

The study is promoted by the Department of Health Sciences of the University of Florence.

The questionnaire is completely anonymous, and data will be collected and managed in aggregated form according to European Union Regulation 2016/679 of European Parliament and the Italian Legislative Decree 2018/101.

Do you agree to participate to the study?

[] I agree [] I do not agree

QUESTIONNAIRE

SECTION 1: SOCIODEMOGRAPHIC INFORMATION

- Age (years): select from 18 to 100

- Gender: [ ] M; [ ] F

- Do you work or have an education in the healthcare sector? [ ] Yes; [ ] No

- Do you have minor offspring? [ ] Yes; [ ] No

SECTION 2: KNOWLEDGE ABOUT VACCINE PRODUCTION PROCESS

- In your opinion, are controls carried out during vaccine production? [ ] Yes; [ ] Not enough; [ ] No

- Compared to the most common drugs, the vaccine production is: [ ] More controlled; [ ] Less controlled; [ ] Equally controlled

- How long does the process of vaccine production take? [ ] Less than 2 months; [ ] 6-24 Months; [ ] 3-5 years

- During the vaccine production process, how much time is spent on quality controls? [ ] <10% of the time; [ ] 10-50% of the time; [ ] >50% of the time

- Who controls the vaccine production process? [ ] National Health Institute; [ ] The drug company; [ ] External laboratories; [ ] All previous answers; [ ] None of the previous answers

- When is the quality of vaccine controlled? [ ] At different stages of production; [ ] During the shipping and the storage; [ ] All the previous answers; [ ] None of the previous answers

- Do you think the vaccine could be contaminated by impurities during production process? [ ] Yes; [ ] No

- Do you think adequate controls are carried out to ensure that the vaccine is not contaminated by impurities during the production process? [ ] Yes; [ ] No

- Is it guaranteed that the licensed vaccine has the same characteristics as the one studied before the production process? [ ] Yes; [ ] No

- If one day you could visit a pharmaceutical industry, what would you like to ask or to see? (Open short answer)

TABLES

Table S1. Results of Section 2 of questionnaire: summary of the distribution of knowledge and opinions about vaccine production process stratified by socio-demographic characteristics. [NOTE. HC: Healthcare. §: correct answer].

		**Female**		**Male**		**Work/education: HC**		**Work/education: not HC**		**Minor offpsring**		**Non-minors offspring**		**All**	
		**N**	**%**	**N**	**%**	**N**	**%**	**N**	**%**	**N**	**%**	**N**	**%**	**N**	**%**
**In your opinion, are controls carried out during the vaccine production?**	**Yes**	83	94.3	44	93.6	47	100.0	80	90.9	35	92.1	92	94.8	127	94.1
	**No**	5	5.7	3	6.4	0	0	8	9.1	3	7.9	5	5.2	8	5.9
	Total	88	100	47	100	47	100	88	100	38	100	97	100	135	100
	p-value	0.87				**0.03**				0.54					
**Compared to the most common drugs, the vaccine production is:**	**More controlled (§)**	35	39.8	25	53.2	20	42.6	40	45.5	14	36.8	46	47.4	60	44.4
	**Less controlled**	3	3.4	1	2.1	0	0	4	4.5	1	2.6	3	3.1	4	3.0
	**Equally controlled**	50	56.8	21	44.7	27	57.4	44	50.0	23	60.5	48	49.5	71	52.6
	Total	88	100	47	100	47	100	88	100	38	100	97	100	135	100
	p-value	0.32				0.28				0.51					
**How long does the process of vaccine production take?**	**Less than 2 months**	6	6.8	3	6.4	2	4.3	7	8.0	3	7.9	6	6.2	9	6.7
	**6-24 months (§)**	61	69.3	28	59.6	32	68.1	57	64.8	20	52.6	69	71.1	89	65.9
	**3-5 years**	21	23.9	16	34.0	13	27.7	24	27.3	15	39.5	22	22.7	37	27.4
	Total	88	100	47	100	47	100	88	100	38	100	97	100	135	100
	p-value	0.45				0.71				0.11					

Table S1.

		Female		Male		Work/education: HC		Work/education: not HC		Minors offpsring		Non-minors offspring		All	
		N	%	N	%	N	%	N	%	N	%	N	%	N	%
**During the vaccine production process, how much time is spent on quality controls?**	**<10% of the time**	3	3.4	2	4.3	1	2.1	4	4.5	2	5.3	3	3.1	5	3.7
	**10-50% of the time**	29	33.0	17	36.2	9	19.1	37	42.0	11	28.9	35	36.1	46	34.1
	**>50% of the time (§)**	56	63.6	28	59.6	37	78.7	47	53.4	25	65.8	59	60.8	84	62.2
	Total	88	100	47	100	47	100	88	100	38	100	97	100	135	100
	p-value	0.89				**0.02**				0.65					
**Who controls the vaccine production process?**	**National Health Institute**	24	27.3	14	29.8	6	12.8	32	36.4	12	31.6	26	26.8	38	28.1
	**The drug company**	7	8.0	2	4.3	5	10.6	4	4.5	1	2.6	8	8.2	9	6.7
	**External laboratories**	7	8.0	3	6.4	4	8.5	6	6.8	4	10.5	6	6.2	10	7.4
	**All the previous answers (§)**	50	56.8	28	59.6	32	68.1	46	52.3	21	55.3	57	58.8	78	57.8
	**None of the previous answers**	0	0.0	0	0.0	0	0.0	0	0.0	0	0.0	0	0.0	0	0.0
	Total	88	100	47	100	47	100	88	100	38	100	97	100	135	100
	p-value	0.84				**0.03**				0.52					

Table S1.

		Female		Male		Work/education: HC		Work/education: not HC		Minors offpsring		Non-minors offspring		All	
		N	%	N	%	N	%	N	%	N	%	N	%	N	%
**When is the quality of vaccine controlled?**	**At all stages of production**	40	45.5	24	51.1	16	34.0	48	54.5	19	50.0	45	46.4	64	47.4
	**During transportation and storage**	1	1.1	0	0.0	1	2.1	0	0.0	0	0.0	1	1.0	1	0.7
	**All the previous answers (§)**	46	52.3	21	44.7	29	61.7	38	43.2	18	47.4	49	50.5	67	49.6
	**None**	1	1.1	2	4.3	1	2.1	2	2.3	1	2.6	2	2.1	3	2.2
	Total	88	100	47	100	47	100	88	100	38	100	97	100	135	100
	p-value	0.49				0.08				0.91					
**Do you think the vaccine could be contaminated by impurities during the production process?**	**Yes**	41	46.6	19	40.4	23	48.9	37	42.0	18	47.4	42	43.3	60	44.4
	**No**	47	53.4	28	59.6	24	51.1	51	58.0	20	52.6	55	56.7	75	55.6
	Total	88	100	47	100	47	100	88	100	38	100	97	100	135	100
	P Value	0.49				0.44				0.67					
**Do you think adequate controls are carried out to ensure that the vaccine is not contaminated with impurities during the production process?**	**Yes**	78	88.6	43	91.5	46	97.9	75	85.2	33	86.8	88	90.7	121	89.6
	**No**	10	11.4	4	8.5	1	2.1	13	14.8	5	13.2	9	9.3	14	10.4
	Total	88	100	47	100	47	100	88	100	38	100	97	100	135	100
	P Value	0.60				**0.02**				0.51					

Table S1.

		Female		Male		Work/education: HC		Work/education: not HC		Minors offpsring		Non-minors offspring		All	
		**N**	**%**	**N**	**%**	**N**	**%**	**N**	**%**	**N**	**%**	**N**	**%**	**N**	**%**
**Is it guaranteed that the licensed vaccine has the same characteristics as the one studied before the production process?**	**Yes**	65	73.9	42	89.4	40	85.1	67	76.1	26	68.4	81	83.5	107	79.3
	**No**	23	26.1	5	10.6	7	14.9	21	23.9	12	31.6	16	16.5	28	20.7
	Total	88	100	47	100	47	100	88	100	38	100	97	100	135	100
	P Value	**0.03**				0.22				**0.05**					

Table S2. Results of Section 2 of questionnaire: answers related to the age groups. [NOTE. §: correct answer].

		20-29 years		30-39 years		40-49 years		>49 years		Total		
		N	%	N	%	N	%	N	%	N	%	*p*-value
**In your opinion, are controls carried out during the vaccine production?**	**Yes**	47	97.9	48	94.1	15	83.3	17	94.4	127	94.1	0.172
	**No**	1	2.1	3	5.9	3	16.7	1	5.6	8	5.9	
**Compared to the most common drugs, the vaccine production is:**	**More controlled (§)**	22	45.8	24	47.1	6	33.3	8	44.4	60	44.4	0.253
	**Less controlled**	0	0.0	2	3.9	0	0.0	2	11.2	4	3.0	
	**Equally controlled**	26	54.2	25	49.0	12	66.7	8	44.4	71	52.6	
**How long does the process of vaccine production take?**	**Less than 2 months**	4	8.3	3	5.9	1	5.6	1	5.6	9	6.7	0.582
	**6-24 months (§)**	35	72.9	29	56.9	12	66.7	13	72.2	89	65.9	
	**3-5 years**	9	18.8	19	37.3	5	27.8	4	22.2	37	27.4	
**During the vaccine production process, how much time is spent on quality controls?**	**<10% of the time**	2	4.2	2	3.9	0	0.0	1	5.6	5	3.7	0.707
	**10-50% of the time**	18	37.5	13	25.5	8	44.4	7	38.9	46	34.1	
	**>50% of the time (§)**	28	58.3	36	70.6	10	55.6	10	55.6	84	62.2	

Table S2.

		20-29 years		30-39 years		40-49 years		>49 years		Total		
		N	%	N	%	N	%	N	%	N	%	*p*-value
**Who controls the vaccine production process?**	**National Health Institute**	12	25.0	14	27.5	4	22.2	8	44.4	38	28.1	0.212
	**The drug company**	5	10.4	3	5.9	1	5.6	0	0.0	9	6.7	
	**External laboratories**	2	4.2	4	7.8	4	22.2	0	0.0	10	7.4	
	**All the previous answers (§)**	29	60.4	30	58.8	9	50.0	10	55.6	78	57.8	
	**None of the previous answers**	0	0.0	0	0.0	0	0.0	0	0.0	0	0.0	
**When is the quality of vaccine controlled?**	**At all stages of production**	19	39.6	24	47.1	9	50.0	12	66.7	64	47.4	0.669
	**During transportation and storage**	1	2.1	0	0.0	0	0.0	0	0.0	1	0.7	
	**All the previous answers (§)**	27	56.3	26	51.0	8	44.4	6	33.3	67	49.6	
	**None of the previous answers**	1	2.1	1	2.0	1	5.6	0	0.0	3	2.2	

Table S2.

		20-29 years		30-39 years		40-49 years		>49 years		Total		
		**N**	**%**	**N**	**%**	**N**	**%**	**N**	**%**	**N**	**%**	***p*-value**
**Do you think the vaccine could be contaminated by impurities during the production process?**	**Yes**	19	39.6	25	49.0	9	50.0	7	38.9	60	44.4	0.719
	**No**	29	60.4	26	51.0	9	50.0	11	61.1	75	55.6	
**Do you think adequate controls are carried out to ensure that the vaccine is not contaminated by impurities during the production process?**	**Yes**	45	93.8	45	88.2	15	83.3	16	88.9	121	89.6	0.623
	**No**	3	6.3	6	11.8	3	16.7	2	11.1	14	10.4	
**Is it guaranteed that the licensed vaccine has the same characteristics as the one studied before the production process?**	**Yes**	41	85.4	40	78.4	12	66.7	14	77.8	107	79.3	0.409
	**No**	7	14.6	11	21.6	6	33.3	4	22.2	28	20.7	
